# Impact of molecular configuration on the photoluminescence and electrical characteristics of poly-pyrrol-thiazol-imine polymers films

**DOI:** 10.1038/s41598-024-79758-5

**Published:** 2024-11-16

**Authors:** Ahmed R. Ghazy, El-Refaie Kenawy, Nourhan Darwesh, S. Shendy, Abdelhamid El-Shaer, R. Ghazy

**Affiliations:** 1 Laser Laboratory, Physics Department, Faculty of Science, Tanta University, Tanta, 31527 Egypt; 2 Polymer Research Group, Department of Chemistry, Faculty of Science, Tanta University, Tanta, 31527 Egypt; 3 Physics Department, Faculty of Science, Kafrelsheikh University, Kafrelsheikh, 33516 Egypt

**Keywords:** *Poly-pyrrol-thiazol-imine*, *Molecular configuration*, *Optical properties*, *Electrochemical spectroscopy*, *Dielectric properties*, Materials science, Optical materials and structures

## Abstract

The optical, photoluminescence, and electrical properties of Poly(Z)-PTI and Poly(E)-PTI, two Poly-Pyrrol-Thiazol-Imine polymers with comparable chemical structures but distinct configurations, were examined. Using the dip-casting method, polymer films were deposited on ITO substrates. UV-VIS spectroscopy revealed that both polymers diverged between 500 and 800 nm, showing the impact of molecular arrangement, but showed similar absorption behavior for wavelengths shorter than 500 nm. For Poly(Z)-PTI, the direct optical energy gaps were 2.06 eV, while for Poly(E)-PTI, they were 1.78 eV. Poly(Z)-PTI displayed an emission peak at 610 nm (red) according to laser photoluminescence spectra, while Poly(E)-PTI peaked at 563 nm (green-yellow). The capacitance behavior was revealed by electrochemical impedance spectroscopy. Nyquist plots suggested an equivalent circuit model of R_s_ (CR_ct_)(QR)(CR) for both polymers, and the relaxation times were 15.9 ns for Poly(Z)-PTI and 89.5 ns for Poly(E)-PTI. The Mott-Schottky analysis verified the n-type conductivity, revealing 2.18 × 10^16^ cm^− 3^ carrier densities for Poly(Z)-PTI and 1.78 × 10^16^ cm^− 3^ for Poly(E)-PTI. At lower frequencies, both polymers exhibited limited conductivity and large dielectric constants. Insights into the possible uses of Poly-Pyrrol-Thiazol-Imine polymers in electrical and optoelectronic devices are provided by this study, which emphasizes the influence of molecular configuration on these polymers’ characteristics.

## Introduction

A class of materials known as conjugated polymers has surfaced, and their extraordinary qualities make them very appealing for use in optoelectronic applications. Conjugated polymers, as opposed to conventional insulating polymers, have extended π-electron systems due to alternating single and double bonds throughout their backbone. The polymers have semiconducting qualities due to their distinct molecular structure, which makes it possible for them to carry electrical charges effectively. These materials show intriguing optical and electrical properties in the field of optoelectronics. Electrons can be more efficiently transported by virtue of the π-conjugation, which also makes them a great option for usage in organic solar cells, organic field-effect transistors (OFETs), and light-emitting diodes (OLEDs)^1,2^.

Due to structural alterations and chemical design, conjugated polymers have customizable electrical properties that give researchers a flexible toolkit to customize their performance for particular uses^3^. This has led to the development of conjugated polymers as a key component in lightweight, flexible, and affordable optoelectronic devices, opening the door for developments in electronic fabrics, flexible displays, and renewable energy^4,5^.

Renowned for their remarkable mechanical strength, thermal stability, chemical inertness, strong electrical conductivity, fluorescence, and photo-acoustic qualities, polyimines are classified as “high-performance” conjugated polymers. Polyimines are positioned as adaptable and promising materials for a range of cutting-edge applications due to their special combination of qualities^6^.

Polyimines are noteworthy for their processability and flexibility, which makes them appropriate for applications where different shape conformance is required^7^. Furthermore, polyimines have a high electrical conductivity, which is good for electronic equipment^8,9^. Their versatility in synthesizing with different functional groups also enables customization of their characteristics for particular uses^10,11^.

Polyimine-based materials have been investigated for use in flexible substrates, organic light-emitting diodes (OLEDs), organic photovoltaics (OPVs), and other flexible electronic devices in the context of optoelectronics and flexible electronics^12^. In the quickly developing field of innovative materials for electronics, polyimine chemistry’s adaptability and capability for solution processing add to its appeal. In the field of optoelectronics, scientists are still exploring and creating new polyimine-based materials with improved performance and characteristics for a variety of uses^13^.

Because of its many uses in materials science, especially in the creation of customized functional materials, polyimine polymers have drawn a lot of attention. We synthesize and characterize two distinct polyimine polymers in this work: Poly(Z)-PTI and Poly(E)-PTI. These polymers are produced via condensation reactions between pyrrole-2,5-dicarbaldehyde and various amines in ethanol. Because these polymers have different cis (Z) and trans (E) orientations around the double bond, they display interesting structural differences^14^. The physical and chemical properties of polymers are influenced by the particular arrangements of substituents, which lead to different molecular geometries^15,16^.

With a Z (cis) configuration, Poly(Z)-PTI exhibits a specific functional group arrangement that provides novel insights into its structural properties and its uses. On the other hand, Poly(E)-PTI, with its E (trans) configuration, offers a different arrangement that results in a distinct collection of characteristics and behaviors. Deciphering how these configurations affect the polymers’ overall structure and reactivity is essential to enabling a wide range of uses, from biomedical materials to optoelectronics^17–19^. The present study explores the synthesis and characterization of Poly(Z)-PTI and Poly(E)-PTI, with the aim of providing insights into their prospective applications in the advancement of polyimine-based materials across many scientific and technical fields.

The diverse qualities of polyimine polymers, such as their mechanical strength, thermal stability, and tunable electrical characteristics, have drawn a lot of attention. According to Dong et al., 2023^20^, a number of investigations have shown the promise of polyimine derivatives in optoelectronic and photoluminescent applications, with improved thermal and optical properties. Furthermore, 2D polymer (2DPI) membranes based on imines have become attractive options for osmotic energy conversion, resolving the conflict between conductivity and ion selectivity. According to Zhang et al., 2022^21^, recent research has demonstrated that 2DPI membranes’ nanometer-scale thickness and ion-selective interactions allow them to record power densities up to approximately 53 W/m² and achieve good Na^+^ selectivity (0.98). The structural variety and photophysical characteristics of polyimines are still poorly understood, nonetheless, in spite of these developments.

Building on our previous work^22^, this work aims to explore the effects of various configurations in polyimine polymers, namely Poly(Z)-PTI and Poly(E)-PTI. After these polymers are synthesized and shaped into polymeric films, their optical, photoluminescence, and electrical characteristics are thoroughly investigated. Through an examination of these physical attributes, the research seeks to clarify how different configurations affect the overall behavior of the polyimine polymers, offering important information about their possible uses and qualities.

## Experimental

### Materials

**Fig. 1:**
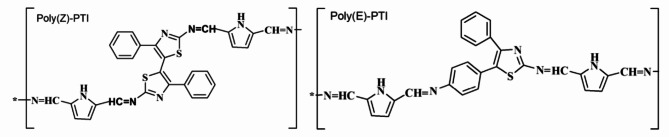
Chemical structure of Poly(Z)-Pyrrol/Thiazol-Imine (Poly(Z)-PTI) and Poly(E)-Pyrrol-Thiazol-Imine (Poly(E)-PTI).

Pyrole-2,5-dicarbaldehyde 1 was reacted with two different amines, 5-(2-amino-4-phenylthiazol-5-yl)-4-phenylthiazol-2-amine 2 and 5-(4-Amino-phenyl)-4-phenylthiazol-2-amine 3, in ethanol to create two polyimine polymers, Poly(Z)-Pyrrol/Thiazol-Imine (Poly(Z)-PTI) and Poly(E)-Pyrrol-Thiazol-Imine (Poly(E)-PTI) Fig. 1. with a IUPAC names of poly(Z)-N-((5-(iminomethyl) -1 H-pyrrol-2-yl)methylene) -5- (2- ((E) -(5- (iminomethyl) -1 H- pyrrol-2-yl) methyleneamino) -4- phenylthiazol-5-yl) -4- phenylthiaol − 2- imine (P1) and poly(E)-N- ((5-(iminomethyl) -1 H- pyrrol − 2-yl) methylene) -5- (4- ((E)- (5- (iminomethyl) -1 H- pyrrol-2-yl) methyleneamino) phenyl) -4- phenylthiaol − 2-imine, respectively. These polyimine polymers display their distinct compositions by containing thiazole and pyrrole units together with imine links (C = N). The polymerization process by normal heating starts after 30 min since it is insoluble in ethanol. The orange and brown precipitates (Poly(Z)-PTI and Poly(E)-PTI) that are produced by this early precipitation are the outcome. The molecular weight of the Poly(Z)-PTI and Poly(E)-PTI was measured using static laser light scattering technique^23–26^ and found to be 5484 and 10,622 g/mol for Poly(Z)-PTI and Poly(E)-PTI respectively. Previous work has documented the complete synthesis and characterizations of the two polymers utilizing FTIR, XRD, TGA, EDX, and 1 H NMR in addition to static light scattering^22^.

### Methodology

In this work, the dip casting method was used to deposit thin films of Poly(Z)-PTI and Poly(E)-PTI onto ITO substrates. In order to create a consistent film, the ITO substrates had to be dipped into a solution containing the corresponding polyimines. The optical characteristics of the films that were deposited were then examined using UV-VIS spectroscopy, which was conducted using a V-630 Jasco spectrophotometer. The absorption and transmission properties of the films may be examined thanks to this method. Furthermore, photoluminescence studies were carried out utilizing a 325 nm He-Cd laser source. Through the use of a computerized CCD camera and a HoRiBA (IHR 320) spectrum analyzer, the light emitted from the polyimine films was captured. Using a CHI660E electrochemical workstation in a three-electrode configuration, the electrochemical properties of the polyimine samples were assessed. The synthetic polyimine derivative was used as the working electrode, while the reference electrode and counter electrode were an Ag/AgCl electrode and a platinum wire, respectively. While the current-voltage (I-V) characteristics were collected by scanning the potential from − 1 to 1 V, the impedance measurements were conducted in the frequency range of 0–10 kHz. Using repeated scans, the samples’ stability was evaluated.

## Results and discussion

### Optical properties

**Fig. 2:**
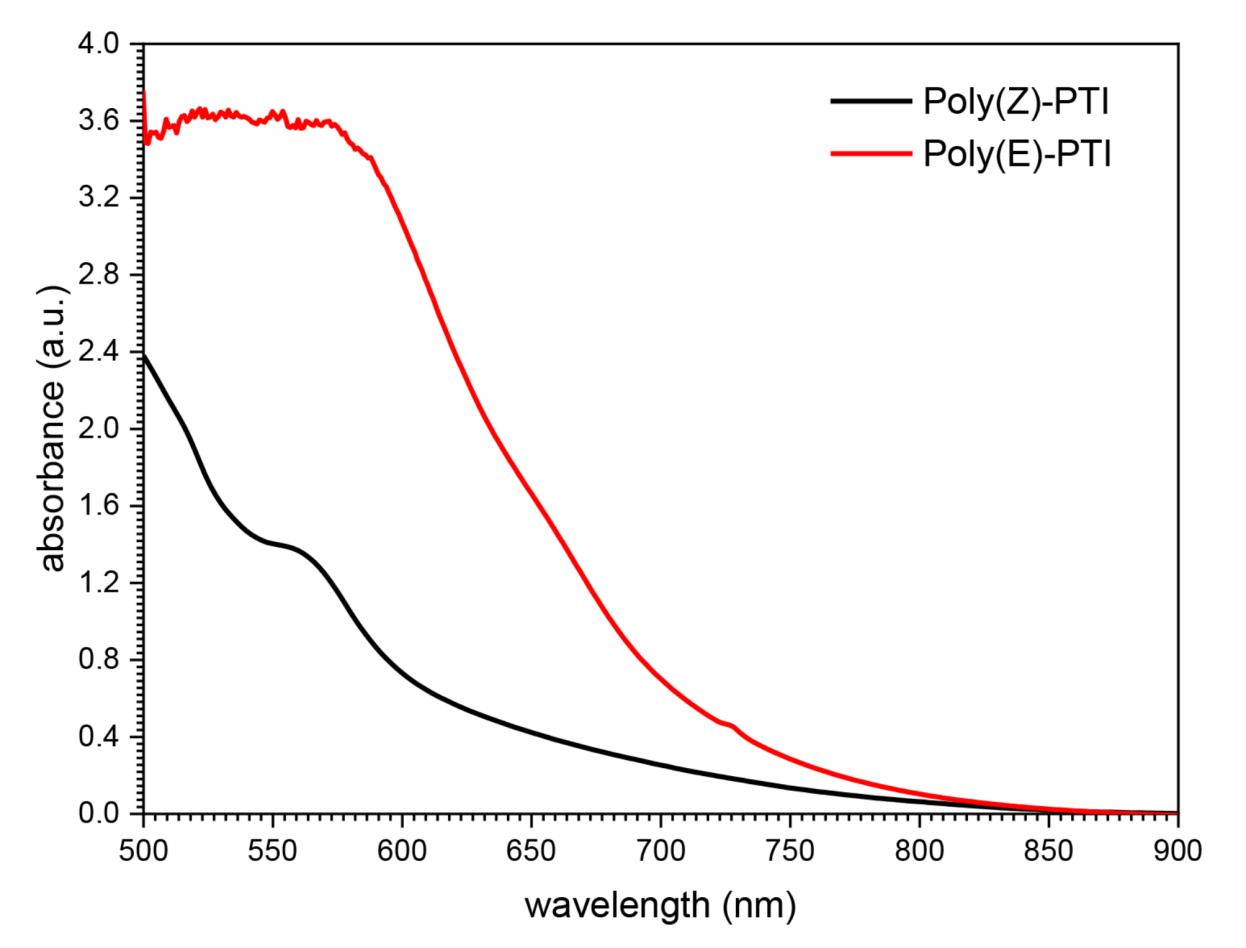
UV-VIS absorbance spectra of Poly(Z)-PTI and Poly(E)-PTI films.

Polyimine is a unique polymer possessing distinct absorption spectra that contributes to its outstanding characteristics. In fact, alterations to polyimine’s chemical structure can significantly affect its absorption spectra. Modifications to the substituents, polymerization conditions, or monomer structure are just a few of the numerous ways that polyimine can be tailored to meet particular demands. The energy levels and electrical configuration of the polymer may be affected by these structural changes, which could result in appreciable changes in the material’s absorption spectra. Figure 2. shows the absorption spectra of the two polyimines Poly(Z)-PTI and Poly(E)-PTI films. Electromagnetic waves shorter than 500 nanometers are absorbed by both polymers. An absorption band seen at 560 nm in polymer Poly(Z)-PTI may be a sign of a potential n-π* transition^27,28^. However, polymer Poly(E)-PTI exhibits a wider absorption range, absorbing all wavelengths below 590 nm. Across a larger range of wavelengths, Poly(E)-PTI notably shows a substantially higher absorption intensity than Poly(Z)-PTI. These results highlight how these polymers’ absorption properties are influenced by their chemical composition.

Through optical estimation, the energy gap (E_g_) between the lowest unoccupied molecular orbit (LUMO) and the highest occupied molecular orbit (HOMO) was determined. The Tauc relation was utilized in this process as described in^29–31^:

$$\:{\left(\alpha\:h\upsilon\:\right)}^{2}=B\left(h\upsilon\:-{E}_{g}\right)$$

**Fig. 3:**
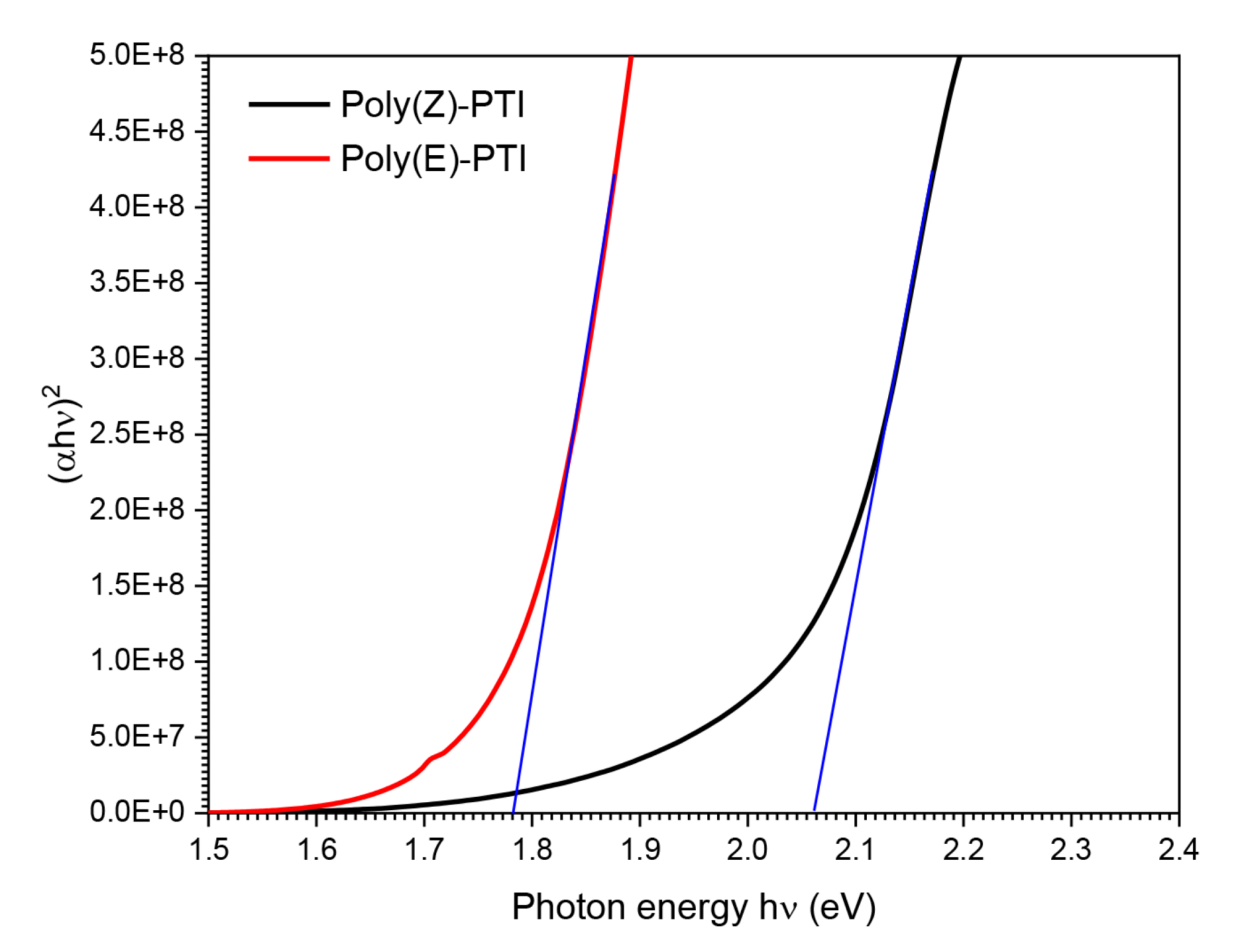
Application of Tauc relation to determine the direct energy gab.

The formula $\:\alpha\:=2.303A/x$ was utilized to computationally determine the absorption coefficient (α), where ‘x’ denotes the sample thickness and hυ denotes the photon energy. Figure 3. provides a graphic representation of this optical method for Poly(Z)-PTI and Poly(E)-PTI films. The intercept between the extrapolated line and the axis was used to calculate the direct optical energy gap. The direct optical energy gap specific values for Poly(Z)-PTI and Poly(E)-PTI were determined to be 2.06 and 1.78 eV, respectively. This in-depth analysis of the energy gap is especially instructive since it clarifies the electronic structure and possible electronic transitions in these polymers. The optical energy gab for the two polymers dissolved in chloroform was measured in the previous work and found to be 2.49 and 2.68 eV for Poly(Z)-PTI and Poly(E)-PTI respectively^22^.

Surprisingly, the measured optical energy gap values agree with published reports for materials frequently used in blue and white light emitting diodes^32,33^. The conformance of Poly(Z)-PTI and Poly(E)-PTI to established values emphasizes its appropriateness for possible optoelectronic device applications, especially in the creation of light-emitting diodes. The thorough optical characterization that is provided here provides important insights into these polymers and their potential applications in cutting-edge optoelectronic and electrical technologies.

**Fig. 4:**
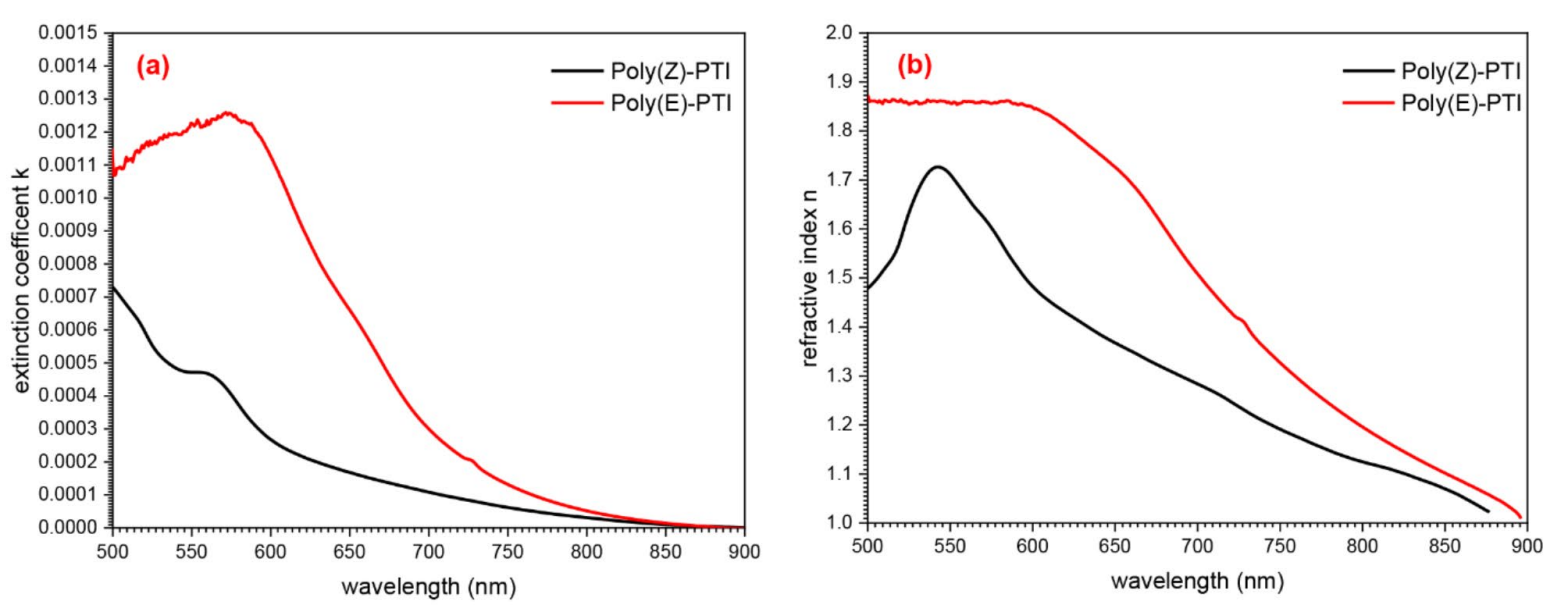
(**a**) extinction coefficient and (**b**) refractive index of Poly(Z)-PTI and Poly(E)-PTI films.

Determining critical parameters, such as the extinction coefficient (k) and refractive index (n) Fig. 4., is crucial for evaluating the optical properties of manufactured films intended for optoelectronic uses. The extinction coefficient (k) was measured using the following formula: $\:k=\frac{\alpha\:\lambda\:}{4\pi\:}\:\:$​, where λ is the wavelength and α is the absorption coefficient. Parallel to this, the refractive index (n) was obtained using the formula $\:n=\left(\frac{1+R}{1-R}\right)+\sqrt{\frac{4R}{{\left(1-R\right)}^{2}}-{k}^{2}}\:$, taking into account reflectance (R), which is calculated as $\:R=1-\sqrt{T.{e}^{A}}$, where T stands for transmittance and A for absorption^34–36^.

**Fig. 5:**
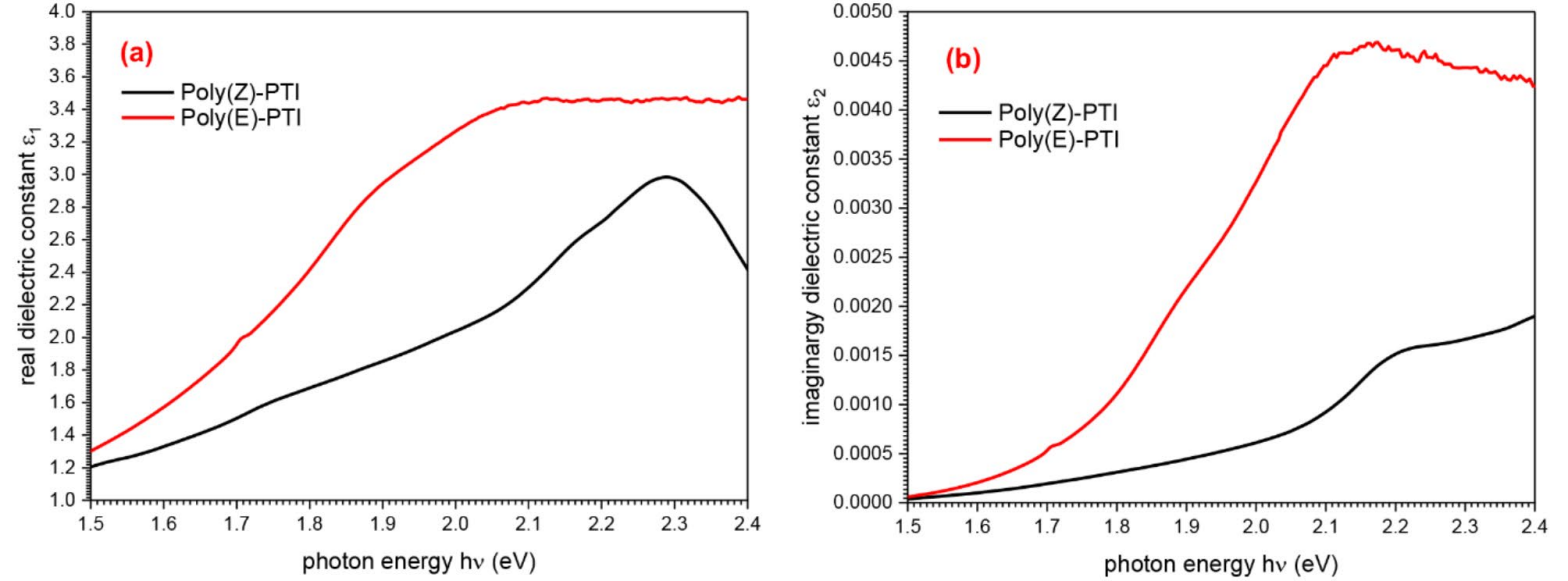
(**a**) real and (**b**) imaginary dielectric constants of Poly(Z)-PTI and Poly(E)-PTI films.

The real (ε_1_) and imaginary (ε_2_) dielectric constants (Fig. 5.) were precisely calculated as part of a thorough examination of the film’s electromagnetic properties^37,38^. The real dielectric constant (ε_1_), which can be obtained using the formula $\:{\epsilon\:}_{1}={n}^{2}-{k}^{2}$, is an important factor in determining how much electromagnetic radiation the film can retain. This actual component provides important information about the material’s viability for applications needing effective wave storage by demonstrating its capacity to absorb and hold electrical energy^39,40^.

Simultaneously, the field losses that the film experiences are revealed by the imaginary dielectric constant (ε_2_), which may be calculated using the formula $\:{\epsilon\:}_{2}=2nk$. The imaginary part of the dielectric constant ε_2_, reveals the energy loss in the material and gives important details about the losses that occur when electromagnetic waves interact with it.

The optical conductivity of the material, denoted by σ(ω), serves as an efficient representation of the substance’s interaction with electromagnetic waves. It is possible to break down this conductivity into its real and imaginary components, which are represented by the symbols σ_1_(ω) and σ_2_(ω), respectively. The following formulas are used to compute these components in terms of the substance’s real (ε_1_(ω)) and imaginary (ε_2_(ω)) dielectric constants^41,42^:

$$\:{\sigma\:}_{1}\left(\omega\:\right)={\epsilon\:}_{o}{\epsilon\:}_{2}\omega\:$$

$$\:{\sigma\:}_{2}\left(\omega\:\right)={\epsilon\:}_{o}{\epsilon\:}_{1}\omega\:$$

**Fig. 6:**
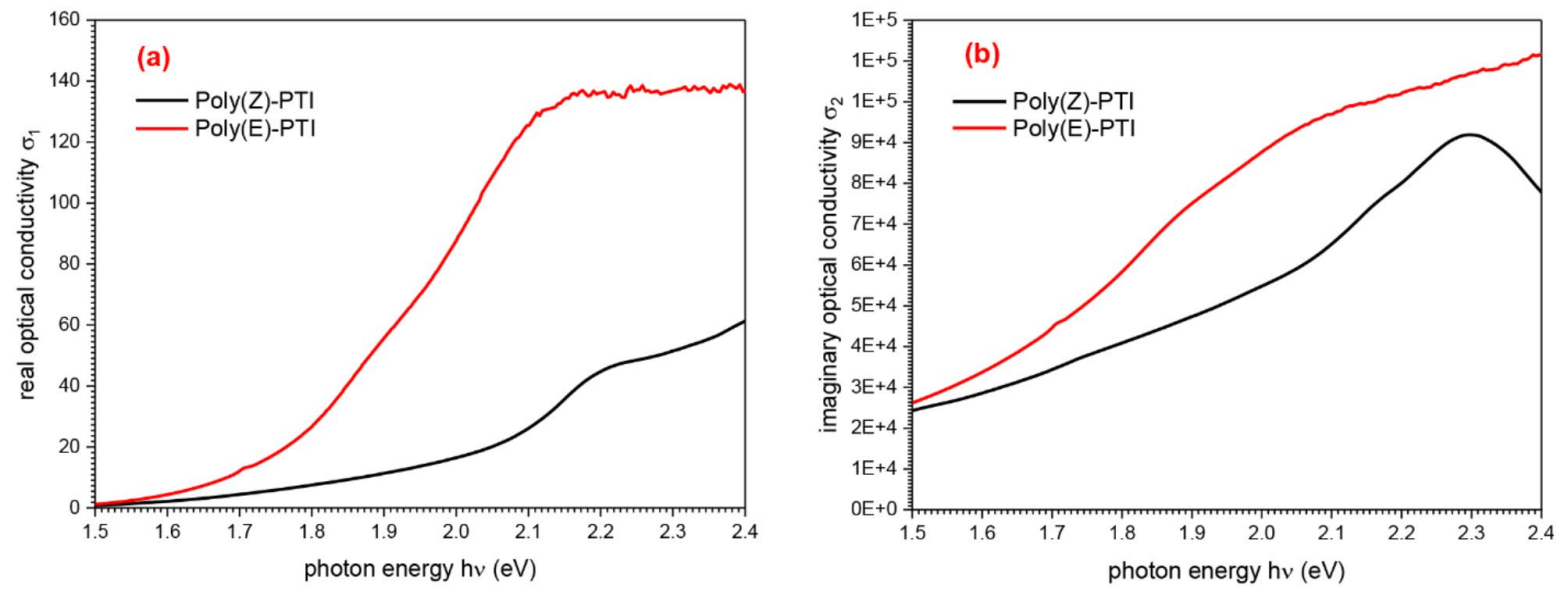
(a) real and (b) imaginary optical conductivity of Poly(Z)-PTI and Poly(E)-PTI films.

In this case, the angular frequency is represented by ω, while the free space permittivity is denoted by ε_o_. Figure 6. shows the consequent fluctuations of σ_1_(ω) and σ_2_(ω) with regard to photon energy.

The impact of the molecular configuration on the optical properties of a conjugated polymer was highlighted by the examination of the optical properties of Poly PTI, which revealed that the Cis configuration had a higher energy gab and a lower refractive index, dielectric constant, and optical conductivity than the Trans configuration.

### Photoluminescence

**Fig. 7:**
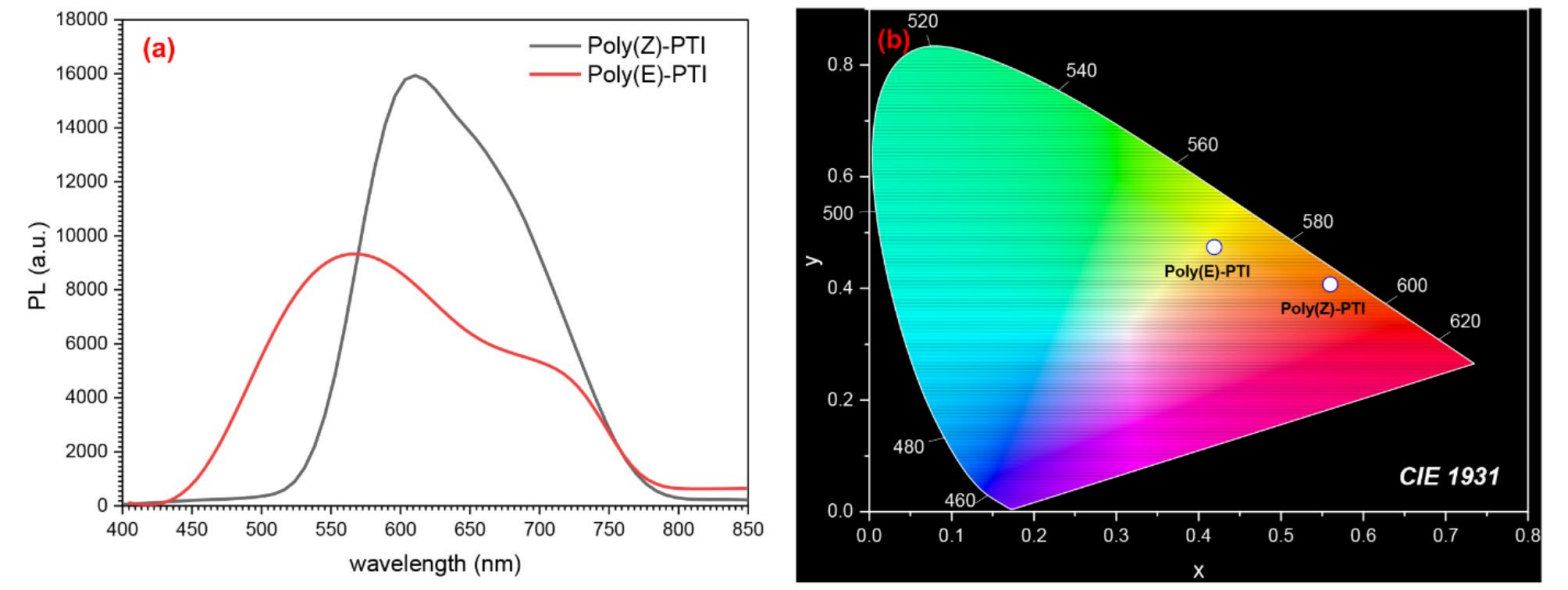
(**a**) laser photoluminescence and (**b**) CIE 1931 color space diagram of Poly(Z)-PTI and Poly(E)-PTI.

The photoluminescence (PL) characteristics and chromaticity coordinates of Poly(Z)-PTI and Poly(E)-PTI, two isomeric polymers with identical chemical structures but different geometric configurations, were investigated Fig. 7 (a and b). According to the PL spectra Fig. 7 (a), Poly(Z)-PTI exhibits high-intensity red emission with a peak PL intensity of about 16,000 a.u. at 600 nm, and lower-intensity green-yellow emission with a peak PL intensity of approximately 7,000 a.u. at 550 nm. This indicates that, in comparison to the E configuration, the Z configuration increases emission strength and shifts it to longer wavelengths. These distinctions are further demonstrated by the CIE 1931 color space diagram Fig. 7 (b), which places Poly(Z)-PTI closer to the red region and Poly(E)-PTI closer to the green-yellow region, highlighting the influence of geometric arrangement on color attributes. The dissimilarities in electronic structure and molecule packing between the Z and E isomers are probably the cause of the fluctuations in PL spectra and chromaticity coordinates. Stronger and red-shifted emission could emerge from the Z configuration’s enhancement of π-π stacking and electronic conjugation, whereas weaker and blue-shifted emission could arise from the E configuration’s fewer effective interactions. Our earlier study determined the quantum yield of photoluminescence for both liquid and solid polymers^22^. Poly(Z)-PTI was discovered to have a quantum yield of 58% in liquid and 59% in solid states. In contrast, the quantum yields for Poly(E)-PTI in the liquid and solid states were 24% and 42.7%, respectively. These findings demonstrate that the CIS arrangement exhibits more photoluminescence properties than the Trans configuration for Poly PTI. By using configurational isomerism to adjust emission characteristics, materials can be designed for specific uses like LEDs, solar cells, and bio-imaging agents.

### Electrical impedance

**Fig. 8:**
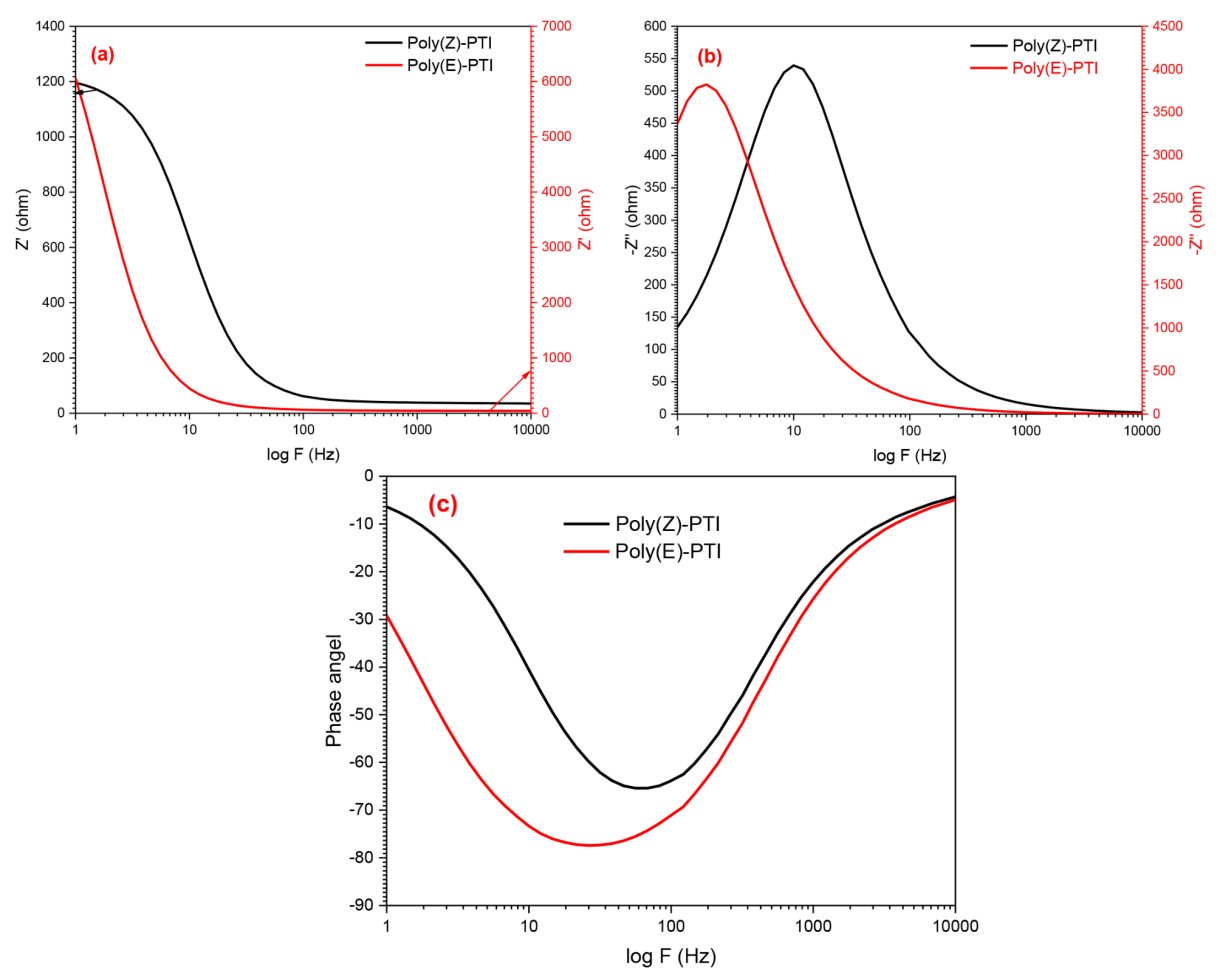
(**a**) real impedance, (**b**) imaginary impedance and (**c**) phase angel of Poly(Z)-PTI and Poly(E)-PTI.

Two polyimines, Poly(Z)-PTI and Poly(E)-PTI, were investigated for their electrical properties and interface behavior using Electrochemical Impedance Spectroscopy (EIS). Through the application of varying frequencies and small amplitude alternating current signals to these polymers, EIS was able to provide important insights into the underlying mechanisms that are essential for the development of biomedical devices, sensors, and energy storage technologies. The real part, imaginary part, and phase angle were the impedance parameters that were shown visually for Poly(Z)-PTI and Poly(E)-PTI in Fig. 8. The behavior, amplitude, and frequency response of the two polymers were found to differ subtly, according to the investigation. Both polymers showed maximal impedance at 1 Hz, with values of 6 kΩ for Poly(Z)-PTI and 1.2 kΩ for Poly(E)-PTI, according to the real component of the electrical impedance Z’. The impedance dropped with increasing frequency, with Poly(E)-PTI exhibiting a sharper fall at lower frequencies. Both polymers had capacitive reactance as shown by negative values of the imaginary part of impedance; maxima could be seen at 2 Hz for Poly(E)-PTI and 10 Hz for Poly(Z)-PTI, which corresponded to distinct capacitive behaviors. In addition, Poly(E)-PTI showed larger Z’’ values than Poly(Z)-PTI, indicating a higher capacitance. The polymer’s capacitive behavior was confirmed by the phase relationship of impedance, which revealed maximum phase differences of -62 at 60 Hz for Poly(Z)-PTI and − 77.5 at 26 Hz for Poly(E)-PTI. This thorough examination advances our knowledge of the electrical characteristics and interface dynamics of Poly(Z)-PTI and Poly(E)-PTI, guiding the best possible application of these materials in a variety of technological contexts.

**Fig. 9:**
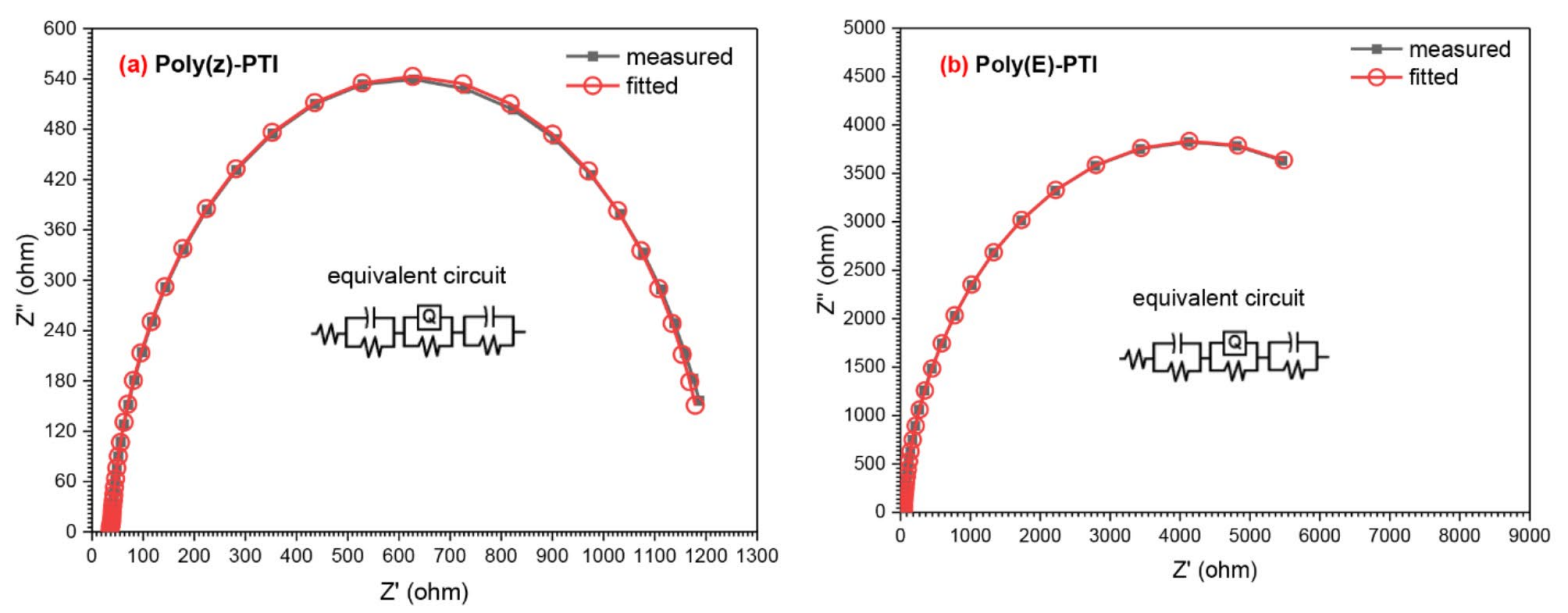
Nyquist plot for (**a**) Poly(Z)-PTI and (**b**) Poly(E)-PTI.

Nyquist (cole-cole) plot was demonstrated by plotting -Z’’ against Z’ for Poly(Z)-PTI and Poly(E)-PTI in order to study the electrical properties of the system, such as the presence of capacitive or inductive behavior, relaxation processes, and interface phenomena. Figure 9. shows the Nyquist plot for Poly(Z)-PTI and Poly(E)-PTI representing the experimental data, fitted curves using ZSimpWin program and the equivalent circuit. Semi-circle behavior was figured out for the two polymers with excellent matching between the experimental and the fitted curves. Both polymers show an equivalent circuit with R_s_ (CR_ct_)(QR)(CR) model. Where R_S_ and R_ct_ represent the solution resistance and charge-transfer resistance, Q is a constant phase element^43^.

Important information about the electrochemical behavior of Poly(Z)-PTI and Poly(E)-PTI materials can be gleaned from the electrical parameters taken out of the Nyquist plot analysis. Interestingly, Poly(E)-PTI has a noticeably greater charge transfer resistance (R_ct_) than Poly(Z)-PTI, suggesting that it is more resistant to charge transfer processes. On the other hand, Poly(Z)-PTI exhibits a greater double-layer capacitance (C_dl_) in comparison to Poly(E)-PTI, indicating a more substantial electrochemical double layer and subsequently increased storage capacity. Additionally, compared to Poly(Z)-PTI, Poly(E)-PTI has a longer relaxation period (τ), suggesting slower response dynamics to perturbations. All of these results, which are summed together in Table 1, point to Poly(Z)-PTI as being more appropriate for applications that require fast charge/discharge cycles and higher capacitance, whereas Poly(E)-PTI would be better suited for situations where slower processes and higher resistance are desirable. These insights help choose materials wisely for a variety of electrochemical applications.

**Table 1:** Summary of Electrical parameters for Poly(Z)-PTI and poly(E)-PTI.

Sample	Z_s_ (Ω)	Z_∞_ (Ω)	*R*_ct_ (Ω)	C_dl_ (µF)	τ (ms)
**Poly(Z)-PTI**	1230	100	1130	14.48	15.9
**Poly(E)-PTI**	8300	100	8200	10.92	89.5

**Fig. 10:**
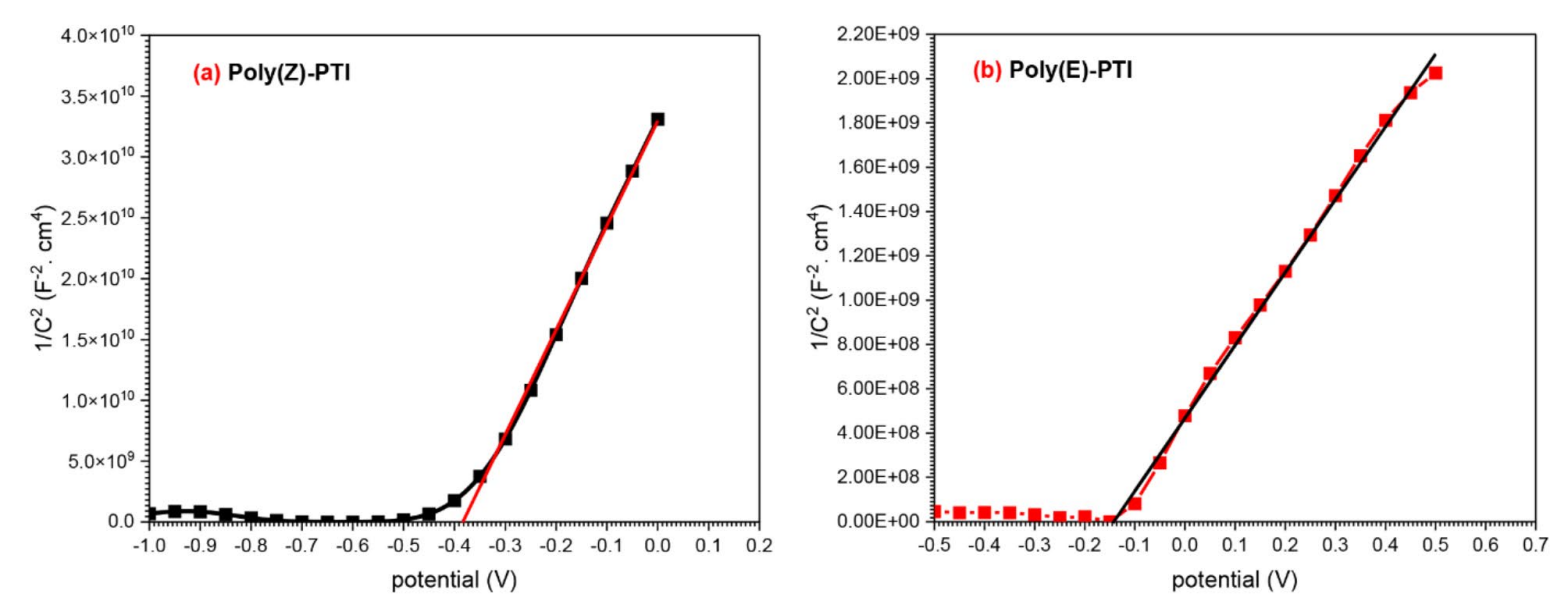
Mott-Schottky plot for (**a**) Poly(Z)-PTI and (**b**) Poly(E)-PTI.

Using electrochemical impedance spectroscopy, the Mott-Schottky analysis was carried out to determine the conductivity type and carrier density in two polymers, Poly(Z)-PTI and Poly(E)-PTI. The space charge capacitance (C) of a Schottky junction is related to the applied voltage (V), carrier concentration (N_A_), flat-band potential (V_fb_), and other factors through the application of the Mott-Schottky equation. The equation is as follows^44,45^:

$$\:\frac{1}{{C}^{2}}=\frac{2}{-\epsilon\:{\epsilon\:}_{o}e{A}^{2}{N}_{d}}\left(V-{V}_{fb}-\frac{kT}{e}\right)$$

where A is the area in contact with the junction, ε is the dielectric constant of the polymers, ε_0_ is the permittivity of vacuum, e is the charge of an electron, k is Boltzmann’s constant, and T is the temperature. From Fig. 10. the V_fb_ values were found to be -0.38 V and − 0.15 V For Poly(Z)-PTI and Poly(E)-PTI, respectively. These results suggest that there is a variance caused by shifts in the band edge positions at the semiconductor-electrolyte interface^46^. Differences in the barrier height and the size of the space charge zone at the interface are shown by this variation in V_fb_. The carrier concentration (N_d_) can be computed by using the slopes from the Mott-Schottky plots of 1/C^2^ vs. V. This yields information on the electronic characteristics of the polymers. The two polymers’ n-type conductivity was demonstrated by their positive slopes, where the carrier densities for Poly(Z)-PTI and Poly(E)-PTI, were calculated to be 2.18 × 10^16^ cm^− 3^ and 1.78 × 10^16^cm^−3^ respectively. According to the Mott-Schottky analysis, there are remarkable carrier densities in Poly(Z)-PTI and Poly(E)-PTI. These high carrier densities imply that there are a significant number of free charge carriers in both polymers, which is suggestive of promising conductive qualities appropriate for electronic applications^47^. Poly(Z)-PTI may have greater conductivity and a better capacity for charge storage because of its marginally higher carrier density. The variations in carrier density demonstrate how molecule structure affects electronic characteristics. These results imply that although both polymers are feasible for use in electronic applications, Poly(Z)-PTI might perform better in situations where greater conductivity is required.

### Dielectric characteristics

**Fig. 11:**
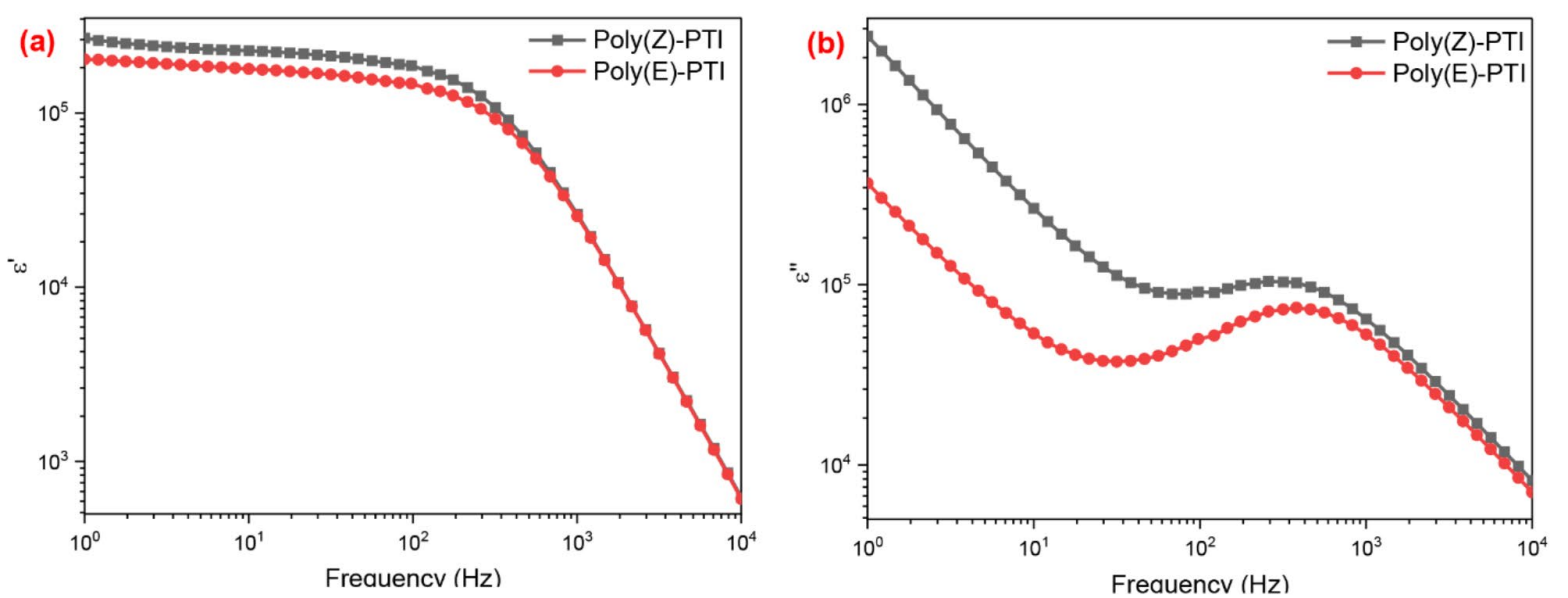
(**a**) real and (**b**) imaginary dielectric constant calculated from impedance spectroscopy for Poly(Z)-PTI and Poly(E)-PTI.

The real and imaginary parts of dielectric constant were obtained from the impedance data in a conventional way using the following relations^48^: $\:{\epsilon\:}^{{\prime\:}}\left(\omega\:\right)=\frac{Z{\prime\:}{\prime\:}}{\omega\:{C}_{o}{\left|Z\right|}^{2}}$ and $\:\epsilon\:{\prime\:}{\prime\:}\left(\omega\:\right)=\frac{Z{\prime\:}}{\omega\:{C}_{o}{\left|Z\right|}^{2}}$, where $\:\left|Z\right|=\sqrt{{Z{\prime\:}}^{2}+{Z{\prime\:}{\prime\:}}^{2}}$. As demonstrated in Fig. 11 (a and b), the real permittivity (ε′) and imaginary permittivity (ε′′) frequency dependencies exhibit a distinctive pattern in dielectric materials, where both ε′ and ε′′ decrease with increasing frequency. The comparatively high values of ε′ indicate that dielectric materials have a high dielectric constant at lower frequencies because of large space charge contributions. Due to the material’s strong permittivity at low frequencies, effective energy storage is made possible by the dipoles’ ability to readily align with the applied electric field^49^. But as the frequency rises, the dipoles fall behind the electric field that is oscillating more quickly, which results in a decrease in ε′. A further decrease in ε′ occurs at very high frequencies, when the dipoles are unable to reorient in synchrony with the field. In a similar vein, the energy dissipation within the material is represented by the imaginary permittivity (ε′′), which likewise exhibits a declining tendency with increasing frequency. Dipolar relaxation and space charge effects are two examples of polarization mechanisms that cause significant energy loss at low frequencies. These mechanisms lose their effectiveness with increasing frequency because the dipoles cannot realign with the changing field quickly enough, which results in lower ε′′ values and less energy dissipation. Understanding and improving the performance of dielectric materials in a variety of electronic and telecommunication applications where their capacity to precisely store and dissipate electrical energy across a range of frequency ranges requires an understanding of this frequency dependent behavior of permittivity.

**Fig. 12:**
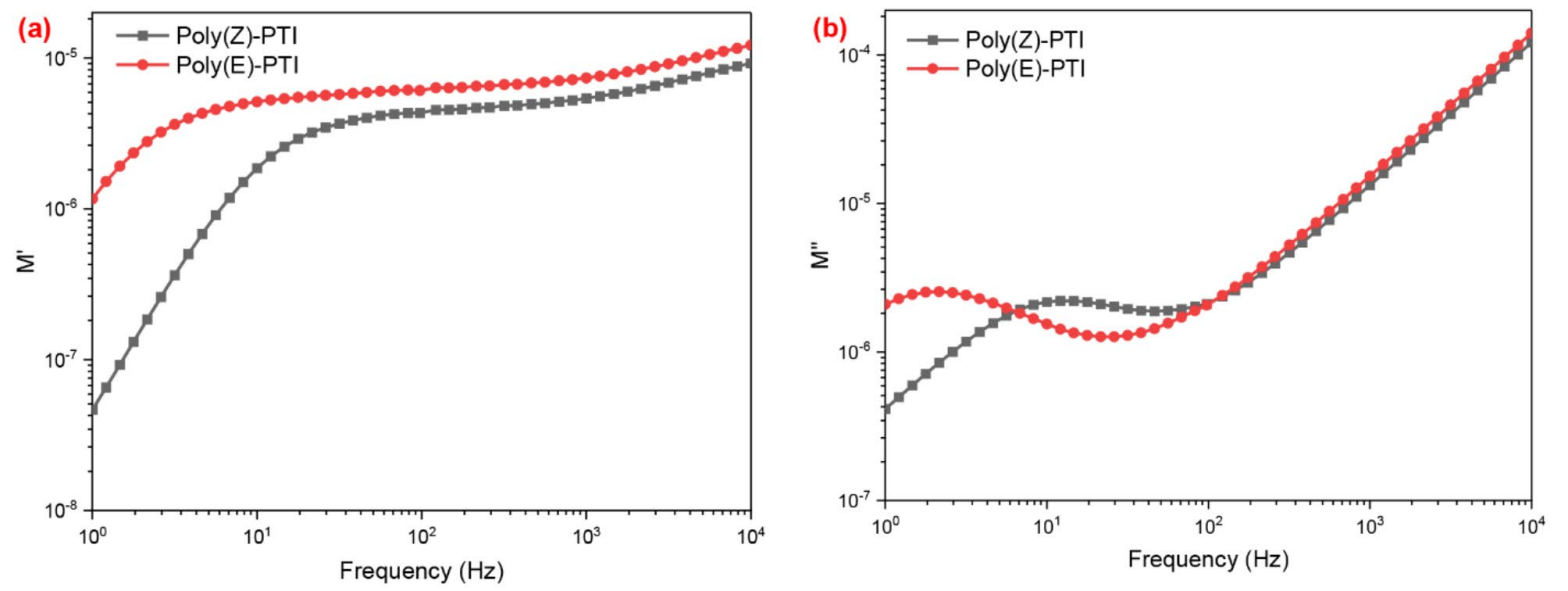
(**a**) real and (**b**) imaginary electric modulus calculated from impedance spectroscopy for Poly(Z)-PTI and Poly(E)-PTI.

Since the modulus formalism successfully suppresses the electrode polarization effects seen at low frequencies, it is an essential method for studying dielectric characteristics. With the use of this formalism, impedance data may be converted into modulus data, which can be written as the complex electric modulus 𝑀*(𝜔), which is inversely related to the complex permittivity ε*(ω). The relationship is expressed as follows^50^: 𝑀*(𝜔) = 1/ ε*(ω), where the complex permittivity and the complex impedance 𝑍*(𝜔) are related by the equation ε*(ω) = 1/(i ωC_o_Z*(ω)). Here, the measuring cell’s vacuum capacitance is denoted by C_o_, and the angular frequency is represented by ω. As a result, the complex modulus can be written as follows: 𝑀*(𝜔) = i ωC_o_Z*(ω). It is possible to divide the complex modulus 𝑀*(𝜔) into its real and imaginary components, 𝑀’(𝜔) and 𝑀’’(𝜔), respectively. These components are calculated from the real and imaginary sections of the impedance, Z′(ω) and Z′′(ω), using the relationships 𝑀’(𝜔)= ωC_o_Z’’(ω) and 𝑀’’(𝜔)= ωC_o_Z’(ω)^51^. By reducing the distorting effects of electrode polarization at low frequencies, the modulus formalism makes it easier to analyze dielectric characteristics accurately. For Poly(Z)-PTI and Poly(E)-PTI, Fig. 12 shows the frequency dependence of the real 𝑀′ and imaginary 𝑀’’ components of the electric modulus, shedding light on their dielectric relaxation behavior. Figure 12a shows the 𝑀′ vs. frequency plot for both polymers, which shows a rising trend with frequency. Interestingly, compared to Poly(Z)-PTI, Poly(E)-PTI continuously exhibits larger 𝑀′ values over the whole frequency range, with a more noticeable difference at lower frequencies that decreases as the frequency increases. Both polymers exhibit distinctive relaxation peaks in the 𝑀’’ vs. frequency plot Fig. 12b, suggesting the existence of dielectric relaxation mechanisms. Compared to Poly(E)-PTI, Poly(Z)-PTI has a peak at a lower frequency, indicating that the two polymers’ relaxation dynamics are different. In addition, Poly(Z)-PTI displays a wider peak than Poly(E)-PTI, which would point to a more intricate relaxation mechanism or a wider range of relaxation durations. These data indicate considerable differences in the dielectric characteristics of the two polymers, with Poly(E)-PTI showing higher values in 𝑀′ and unique relaxation behavior in 𝑀’’. This comparative study highlights how useful the modulus formalism is for differentiating between Poly(Z)-PTI and Poly(E)-PTI’s dielectric behavior, which is essential for customizing materials for certain dielectric applications.

**Fig. 13:**
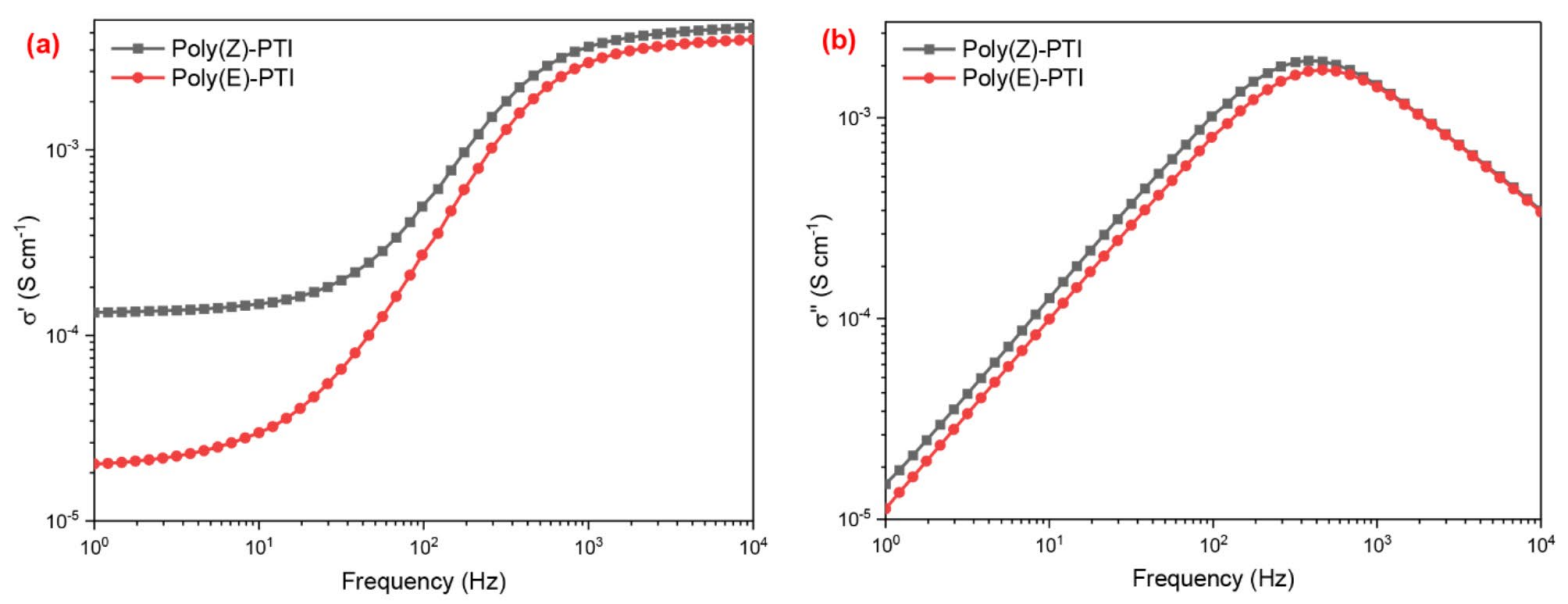
(**a**) real and (**b**) imaginary electrical conductivity calculated from impedance spectroscopy for Poly(Z)-PTI and Poly(E)-PTI.

Because it emphasizes the dynamics of charge carriers at various frequencies, the conductivity formalism is crucial for comprehending the transport characteristics of materials. With this method, impedance data is transformed into conductivity data, which is represented by the complex conductivity σ*(ω). The complex impedance 𝑍*(𝜔) and this conductivity are inversely correlated, according to the formula 𝜎*(𝜔) = 1/Z*(𝜔). By applying the formulas 𝜎’= ωε_o_ε’’ and 𝜎’’= ωε_o_ε’, one may get the real and imaginary components of the complex conductivity Fig. 13^52^. To be more precise, the in-phase component of conductivity, designated as 𝜎’, corresponds to energy dissipation, and the out-of-phase component, designated as 𝜎’’, corresponds to energy storage. Through thorough insights into the mechanics of charge transport and polarization at different frequencies, this transformation enables a more accurate characterization of transport parameters.

The impact of the molecular configuration on the electrical properties of a conjugated polymer was highlighted by the electrical studies, which revealed that the Cis configuration of Poly PTI had a shorter relaxation time, higher carrier density, higher dielectric constant, lower electrical modulus, lower impedance, and higher optical conductivity than those of the Trans configuration. These findings were in line with the optical studies.

## Conclusion

This work thoroughly examines how molecular configuration affects the optical and electrical characteristics of two Poly-Pyrrol-Thiazol-Imine polymers with different configurations but identical chemical structures: Poly(Z)-PTI and Poly(E)-PTI. According to the investigation, Poly(E)-PTI displays a lower energy gap of 1.78 eV with a peak emission at 563 nm, whereas Poly(Z)-PTI displays a direct optical energy gap of 2.06 eV with an emission peak at 610 nm. The different molecular configurations, which also influence the polymers’ photoluminescent properties, are responsible for these variations in optical behavior. Moreover, electrochemical impedance spectroscopy sheds light on both polymers’ capacitive characteristics. With relaxation times of 15.9 ns for Poly(Z)-PTI and 89.5 ns for Poly(E)-PTI, the Nyquist plots show an analogous circuit model of R_s_ (CR_ct_)(QR)(CR), indicating the faster charge transport dynamics in Poly(Z)-PTI. Both polymers’ n-type conductivity is confirmed by Mott-Schottky analysis, which also shows their potential as electron-conducting materials with carrier densities of 2.18 × 10^16^ cm^− 3^ for Poly(Z)-PTI and 1.78 × 10^16^ cm^− 3^ for Poly(E)-PTI. Both polymers have high dielectric constants and restricted conductivity at lower frequencies, suggesting that they are suitable for use in energy conversion and storage devices.

The study shows that the optical and electrical properties of poly PTI are strongly influenced by its molecular structure. In particular, when compared to the Trans configuration, the Cis configuration shows a lower refractive index, dielectric constant, optical conductivity, and a larger energy gap. In contrast, the Cis arrangement has a greater dielectric constant, a lower electrical modulus, a lower impedance, a larger carrier density, a shorter relaxation time, and improved optical conductivity, according to electrical research. These results clearly show a relationship between conjugated polymer performance characteristics and molecular configuration, highlighting the significance of structural design in maximizing material qualities for a range of applications.

## Data Availability

All data generated or analyzed during this study are included in this published article.
